# Complete Genome Sequences of Two *Staphylococcus aureus* Sequence Type 5 Isolates from California, USA

**DOI:** 10.1128/genomeA.00099-17

**Published:** 2017-03-30

**Authors:** Samantha J. Hau, Darrell O. Bayles, David P. Alt, Tracy L. Nicholson

**Affiliations:** a Department of Veterinary Diagnostic and Production Animal Medicine, College of Veterinary Medicine, Iowa State University, Ames, Iowa, USA; b National Animal Disease Center, Agricultural Research Service, USDA, Ames, Iowa, USA

## Abstract

*Staphylococcus aureus* causes a variety of human diseases ranging in severity. The pathogenicity of *S. aureus* can be partially attributed to the acquisition of mobile genetic elements. In this report, we provide two complete genome sequences from human clinical *S. aureus* isolates.

## GENOME ANNOUNCEMENT

*Staphylococcus aureus* is a commensal of the skin and nasopharynx of various animals, including humans. It is also pathogenic in humans, causing disease that ranges in severity from mild skin infections to severe invasive infections (1). Methicillin-resistant *S. aureus* (MRSA) isolates are categorized epidemiologically into three categories: hospital-acquired MRSA (HA-MRSA), community-acquired MRSA (CA-MRSA), or livestock-associated MRSA (LA-MRSA). They are further characterized through multilocus sequence typing into sequence types (STs), which indicate the genetic lineage and characteristics of the isolates. ST5 isolates are widely distributed and known to readily acquire mobile genetic elements containing virulence factors or antibiotic resistance elements (2).

We sequenced two clinical ST5 isolates from the University of California, Irvine (UCI28 and UCI62) (3). They were obtained from patients with MRSA-related disease who had no known exposure to livestock. Because a full patient history was not obtained, HA- and CA-MRSA could not be differentiated. Each isolate was grown in Trypticase soy broth (BD Biosciences, Sparks, MD), and total genomic DNA was extracted using the High Pure PCR template preparation kit (Roche Applied Science, Indianapolis, IN).

Whole-genome sequencing was performed on both the PacBio and Illumina MiSeq platforms. Library preparation for PacBio sequencing was performed according to the PacBio 10-kb insert library preparation protocol available at http://www.pacb.com/wp-content/uploads/2015/09/Procedure-Checklist-10-kb-Template-Preparation-and-Sequencing.pdf. The 10-kb library was sequenced using the PacBio RSII platform, with one single-molecule real-time (SMRT) cell for each isolate. Indexed libraries for the MiSeq protocol were generated with the Nextera XT DNA sample preparation and index kits (Illumina, San Diego, CA), pooled, and sequenced using the MiSeq version 2 500-cycle reagent kit, yielding 2 × 250-bp paired-end reads on the Illumina MiSeq platform (Illumina).

Whole-genome assemblies were generated using PacBio SMRT Analysis version 2.3.0 and the CANU version 1.3 software. The average PacBio coverages for the assembled genomes were 306× for UCI28 and 297× for UCI62. After assembling the PacBio data, any overlapping sequence was trimmed and the genomes oriented to start at the *dnaA* gene. The genomes were polished and error corrected using the Broad Institute’s Pilon program version 1.18, with Illumina data at 75× and 112× average coverage for UCI28 and UCI62, respectively.

### Accession number(s).

The whole-genome sequences for these isolates were deposited in DDBJ/ENA/GenBank with the accession numbers CP018768 and CP018769 for UCI28 and CP018766 and CP018767 for UCI62.
